# A chitosan-lasso peptides nanoparticle for enhanced antibacterial activity and fresh-keeping efficacy in eggs and chilled chicken

**DOI:** 10.1016/j.crfs.2026.101448

**Published:** 2026-05-25

**Authors:** Jinyu Zhang, Yilin Yuan, Huilin Chen, Qianxi Duan, Xinjie Luo, Yong Xiang, Weisheng Cao, Yu Li

**Affiliations:** a College of Veterinary Medicine, South China Agricultural University, Guangzhou, China; b Key Laboratory of Zoonosis Prevention and Control of Guangdong Province, China; c Key Laboratory of Zoonosis of Ministry of Agriculture and Rural Affairs, Guangzhou, China; d Key Laboratory of Veterinary Vaccine Innovation of the Ministry of Agriculture and Rural Affairs, Guangzhou, China; e National and Regional Joint Engineering Laboratory for Medicament of Zoonosis Prevention and Control, China; f Key Laboratory for prevention and control of Avian Influenza and Other Major Poultry Diseases, Ministry of Agriculture and Rural Affairs, China; g Guangdong Province Key Laboratory of Livestock Disease Prevention, China

**Keywords:** Food surface, Nanocoating, *Salmonella* control, Antimicrobial activity, Food preservation

## Abstract

Chemical disinfectants and bacteriophages for controlling *Salmonella* in foods often have limitations such as residual odor, narrow antimicrobial spectra, and poor stability under food processing conditions. Lasso peptides, with their structural stability, heat resistance, and safe antimicrobial activity, are promising candidates for food preservation. In this study, Chitosan-Citric acid-MccJ25-MccY (CCJY) nanoparticles were prepared via citric acid crosslinking to encapsulate MccJ25 and MccY. The nanoparticles showed high encapsulation efficiency (>96.67%) and maintained their antimicrobial activity in the presence of food matrices. CCJY exhibited strong anti-*Salmonella* activity on the surface of eggs and chilled chicken, achieving 99.9% bacterial reduction. Importantly, CCJY retained its efficacy after heat treatment and under acidic/alkaline conditions typical of food processing. In egg preservation, CCJY treatment maintained eggshell integrity (0.82% weight loss, Haugh unit 61.12 after 20 days). In chilled chicken, CCJY reduced total bacterial counts by 2.37 log_10_ CFU/g and decreased drip loss by 12.5% (*p* < 0.001), extending shelf life by 3–4 days. These results demonstrate that CCJY is an efficient and safe antimicrobial formulation suitable for application in egg and meat preservation.

## Highlights

• Innovative CCJY with >90% encapsulation efficiency for MccJ25/MccY and good biocompatibility.
• CCJY shows superior anti-*Salmonella* (MIC: 2–6.25 μg/mL) and stable efficacy under heat/pH stress.
• CCJY inactivates 99.9% of *Salmonella* on eggs and chilled chicken, extending shelf life by 3–4 days.
• CCJY inhibits chilled meat spoilage bacteria (*Pseudomonas*) and maintains food quality.

## Graphical abstract

**Figure:**
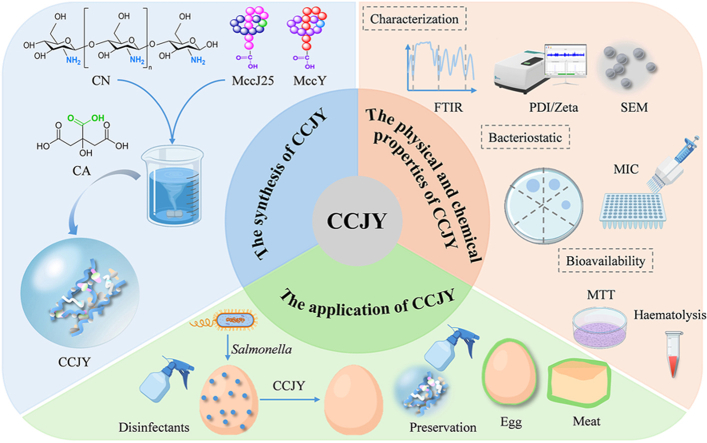

## 1 Introduction

*Salmonella* is a zoonotic pathogen infecting both humans and animals, posing a serious threat to food safety and public health (Eng et al., 2015). According to statistics, this pathogen causes approximately 153 million cases of gastroenteritis and 57,000 deaths annually (Cardoso et al., 2021) Among the more than 3000 identified *Salmonella* serotypes, *Salmonella* Enteritidis (SE) and *Salmonella* Typhimurium (ST) are the two predominant serotypes responsible for human foodborne illnesses (Xiao et al., 2024). They are commonly found in animal-derived foods such as egg products and chilled meat and represent critical sources of contamination and transmission routes (Gu et al., 2024; Liu et al., 2017). However, current control strategies rely primarily on traditional chemical disinfectants and are limited by issues such as antimicrobial resistance and negative impacts on product quality (Sun et al., 2022). Biological agents such as bacteriophages offer specific bactericidal mechanisms but often suffer from narrow antimicrobial spectra, limiting their efficacy against other Gram-positive and Gram-negative bacteria (Huang et al., 2024; Mohammadi et al., 2022). Therefore, there is a need to develop novel broad-spectrum antimicrobial preservatives for food preservation applications.

Composite or biopolymer-based delivery systems have emerged as a promising strategy to enhance the stability and broaden the applicability of antimicrobial agents. Chitosan, a natural polysaccharide with good biocompatibility, film-forming ability, and antimicrobial properties, has been widely used as a food coating and preservation matrix (Wang et al., 2018). For example, a composite film prepared by crosslinking polyvinyl alcohol and citric acid (CA) with chitosan demonstrated structural stability, indicating its potential as a food packaging and preservation material (Jiang et al., 2023). However, the limited bactericidal efficacy of chitosan alone makes it difficult to meet the requirements for highly efficient pathogen control in complex food matrices. Microcins (Mcc), especially lasso peptides with rigid topological structures and excellent physicochemical stability, exhibit potent inhibitory effects against foodborne pathogens (Telhig et al., 2022; Hegemann et al., 2015; Simons et al., 2025). MccJ25, a low-molecular-weight (2 kDa) lasso peptide, exhibits highly efficient bactericidal activity against SE with a minimum inhibitory concentration (MIC) of 2–50 nM but is not effective against ST (Yu et al., 2018). MccY, a recently discovered lasso peptide with a molecular weight of 2 kDa, can kill ST and *S.* Infantis, among other strains, at a concentration as low as 0.125 μM (Li et al., 2021). MccJ25 and MccY have complementary bactericidal properties in different serotypes of *Salmonella*. Notably, free lasso peptides are prone to degradation in complex food matrices, and no studies have reported their combined use for broad-spectrum *Salmonella* control. Furthermore, Citric acid (CA), a food-grade crosslinking agent recognized by the FDA, can mediate stable covalent bonding between chitosan and peptides (Yu et al., 2022), addressing the low loading stability of free peptides. Thus, combining these two complementary lasso peptides with chitosan via CA crosslinking is a promising strategy to develop a safe, broad-spectrum antimicrobial preservative that integrates pathogen control and food preservation.

We hypothesized that citric acid (CA)-mediated crosslinking of chitosan with the lasso peptides MccJ25 and MccY through amide bonds would yield a stable nanocomposite with synergistic antimicrobial and preservative functions. Specifically, the CA-crosslinked chitosan matrix could protect the peptides from degradation, while the combination of MccJ25 and MccY, which possess complementary antibacterial spectra, would enable broad-spectrum bactericidal activity. Moreover, the film-forming properties of chitosan may further support the dual application of this nanocomposite as both an antimicrobial agent and a preservative coating for perishable foods such as eggs and chilled meat. To test this hypothesis, this study aimed to develop novel nanoparticles Chitosan-Citric acid-MccJ25-MccY (CCJY) based on chitosan and lasso peptides, achieving stable loading of MccJ25 and MccY via CA-mediated covalent crosslinking. We systematically characterized CCJYs, and evaluated and compared its antimicrobial efficacy and preservative performance with those of *Salmonella* phages on the surfaces of eggs and chilled meat after spray application. These findings are expected to provide technical support for developing an integrated solution to control foodborne pathogens and ensure food quality.

## 2 Materials and methods

### 2.1 Reagents

Lasso peptides MccJ25 and MccY were prepared using a recombinant expression system previously established in our laboratory (Li et al., 2021, 2024). Briefly, after fermentation, the supernatant was collected by centrifugation, and the target peptides were enriched via C18 solid-phase extraction (Waters, 6 mL/1 g) and eluted with methanol (Li et al., 2023; Gu et al., 2025a). Reversed-phase HPLC was applied for further purification on an Agilent Pursuit XRs C18 preparative column (250 × 21.2 mm, 10 μm), using a 20 min linear gradient of acetonitrile-water (10-50% with 0.1% formic acid) at 21 mL/min. The purified products were identified by UPLC-Q-TOF MS (Agilent 1290-6540) and lyophilized. Both peptides attained over 96% purity, which met the standards for subsequent use.

Chitosan (CN, Mw 161.16 kDa, DD 95%, Sigma-Aldrich, Darmstadt, Germany), glacial acetic acid, Citric Acid (CA, ≥99.5%, Macklin, China), dialysis membrane (MWCO 3 kDa), thiazolyl blue tetrazolium bromide (MTT), Triton X-100, glacial acetic acid, Dulbecco's modified Eagle's medium, fetal bovine serum (Gibco Thermo Fisher Technologies), penicillin-streptomycin (Gibco Thermo Fisher Technologies), bacteriophages CKT1, ФPLL1, and ФF118P13 were used in this study. All the chemicals were of analytical grade.

### 2.2 Lasso peptide purification and CCJY nanoparticle preparation

**Fig. 1:**
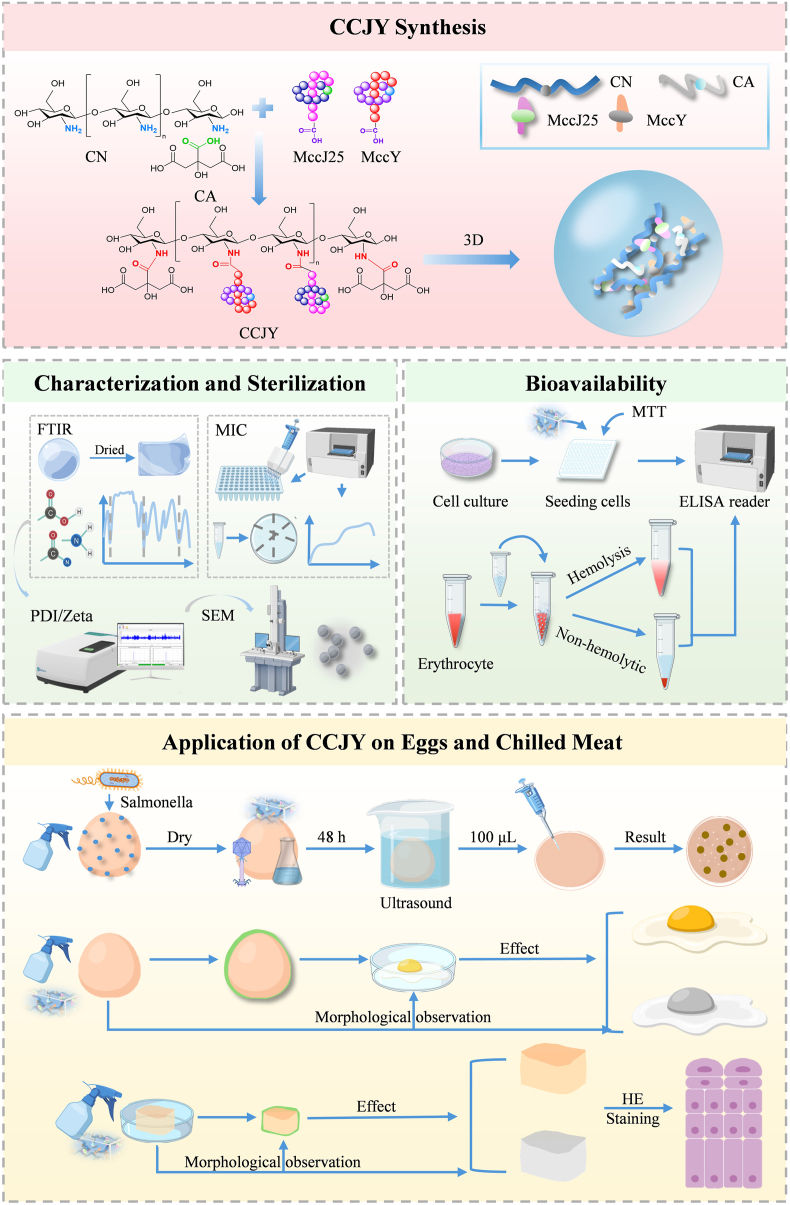
Schematic diagram of the design, synthesis, functional characterization and preservation application of CCJY.

Chitosan was dissolved in 1% (v/v) glacial acetic acid to obtain a final concentration of 1% (w/v, equivalent to 0.01 g/mL) (do Amaral Sobral et al., 2022). The solution was stirred at room temperature until fully dissolved. Separately, lyophilized powders of MccJ25 and MccY (purity:96.2∼97.5%) were each dissolved in sterile deionized water at a concentration of 1 mg/mL. Equal volumes of the two peptide solutions were added to the chitosan solution, and the mixture was stirred at 300 rpm for 2 h to ensure homogeneity. Citric acid (CA, purity≥99.5%) was then added dropwise as a crosslinking agent to a final concentration of 3% (w/v). The resulting mixture was ultrasonication in a cleaning bath (40 kHz, 25 ± 1 °C) for 30 min to promote crosslinking between chitosan and the peptides, facilitating the formation of a nanostructured network. This process yielded an encapsulation efficiency exceeding 96.67% (w/w) in the final nanocarrier. A schematic representation of the synthesis route is provided in Fig. 1.

### 2.3 Characterization of CCJY

Fourier transform infrared spectroscopy (FTIR) was performed in attenuated total reflection mode (Du et al., 2025). The sample films were cut and placed directly on the attenuated total reflection crystal surface. Parameters were set as follows: scanning range of 4000-400 cm^−1^, resolution of 4 cm^−1^, and 32 scans, to obtain the infrared spectra of CCJY and its derivative films (Man et al., 2024; Madhusha et al., 2020). The hydrodynamic diameters and zeta potentials of the synthesized compounds were measured at 25 °C using a Malvern Zetasizer Nano ZS 90 (Malvern Panalytical, UK), an instrument with a measurement range of 0.3 nm-5000 nm.

### 2.4 Assessment of antimicrobial activity and growth curves

The encapsulation efficiency (EE) of CCJY was determined by HPLC. After nanoparticle preparation, the external dialysate was collected, filtered, and analyzed using an Agilent 1260 Infinity II system equipped with a Zorbax SB-C18 column. Peptide concentrations were calculated against a standard curve (r^2^ > 0.999), and EE was calculated as: EE (%) = [(Total drug-Free drug)/Total drug]×100% (Yang et al., 2022; Jafernik et al., 2023). The stability of CCJY was assessed by evaluating its antimicrobial activity via inhibition zone assays after exposure to high temperature (95 °C, 2 h) and extreme pH conditions (pH 2.0 and 12.0, 2 h). Minimum inhibitory concentrations (MICs) against various bacterial strains were determined using the microbroth dilution method (Wang et al., 2024; Kong et al., 2024). The combined effect of the two lasso peptides was evaluated by calculating the fractional inhibitory concentration index (FICI). Interactions were classified based on FICI values: synergy (FICI≤0.5), additive effect (0.5<FICI≤1), indifference (1<FICI≤2), and antagonism (FICI>2). Dynamic bactericidal activity was assessed by continuously monitoring bacterial growth (OD_600_) for 72 h using a Bioscreen C growth curve analyzer. Additionally, the effect of CCJY on *Salmonella* cell membrane integrity was observed using scanning electron microscopy (SEM). All experiments were performed in triplicate and repeated independently three times.

### 2.5 Biocompatibility evaluation

Hemolysis assay: chicken red blood cells were mixed with CCJY and the other compounds at a 1:1 vol ratio, with Triton X-100 and PBS serving as positive and negative controls, respectively. After incubation at 37 °C for 45 min and centrifugation, the absorbance at 540 nm was measured to calculate the hemolysis rate (Gao et al., 2022). MTT Assay for cell metabolic activity: DF-1 cells were cultured for 24 h, then treated with different concentrations of the compounds for another 24 h(Xie et al., 2024). 10 μL of MTT solution (5 mg/mL) was added to each well, followed by incubation for 3.5 h. The culture medium was discarded, and 110 μL of dimethyl sulfoxide was added to each well (Zhao et al., 2022; Gu et al., 2025b). The absorbance of each well was measured at 570 nm using a microplate reader (Lv et al., 2024). Cell morphology observation: morphological changes in the cells after different treatments were observed using a fluorescence inverted microscope (Nikon Corporation). The cells were washed with PBS and directly examined under a microscope using a 20× objective lens. Their morphologies and aggregation states were recorded, and images were captured (Sinha et al., 2021).

### 2.6 Microscopic morphology observation

CCJY nanoparticles were vortexed and dispersed, and 20 μL of the suspension was dropped onto a copper grid. The samples were negatively stained with phosphotungstic acid for 3 min, and their morphology and particle size were observed using a Hitachi HT7700 and SU-8010 transmission electron microscope (TEM) (Ghiasi et al., 2021). Additionally, eggshells stored for 1, 10, and 20 days were dried at 37 °C and trimmed into 1 cm^2^ samples. After sputter coating with platinum, the surface and cross-sectional morphologies of the eggshells were observed using scanning electron microscopy at an accelerating voltage of 15 kV (Bonilla and Sobral, 2016; Sun et al., 2024).

### 2.7 Bactericidal and preservation efficacy of CCJY on eggshell surfaces and eggs

Fresh eggs purchased from Sam's Club (Guangzhou Tianhe Branch, China)were disinfected with 75% ethanol and UV irradiation for 30 min. The sterile eggs were then artificially contaminated with a mixture of SE63 and ST53 (1.0 × 10^8^ CFU/mL). Subsequently, eggs were divided into the following treatment groups: the CCJY group (50, 25, and 12.5 μg/mL), the bacteriophage group (CKT1, ФPLL1, and ФF118P13 at 8.0 × 10^10^ PFU/mL), the CNCA control group, and the PBS control group. Each egg was spray-treated with 0.125 mL of the respective solution and stored at 25°C(Sun et al., 2024). Following the method described by Tu (Tu et al., 2024), samples were collected at 1, 6, 12, 24, 36, and 48 h post-treatment, and viable bacterial counts in the egg wash solution were determined. For preservation evaluation, fresh eggs without any disinfection or bacterial contamination were directly treated with CCJY or CNCA control by spraying 0.125 mL per egg and stored at 25 °C. Samples were taken on days 0, 5, 10, 15, and 20, and the following quality parameters were measured: sensory indicators (scored by three trained panelists according to Supplementary Table S1), weight loss rate [(initial weight − weight at sampling)/initial weight × 100%], Haugh unit: HU = 100 × log(h + 7.57 − 1.7 × w^0.37), yolk index (yolk height/yolk diameter), and albumen index (thick albumen mass/total albumen mass) (Caner and Yüceer, 2015).

### 2.8 Bactericidal and efficacy of CCJY on chilled chicken

For bactericidal efficacy assessment, chilled chicken from Sam's Club was cut into 1 g pieces, surface-sterilized by sequential treatment with 50 mg/L sodium hypochlorite for 30 s, rinsed with sterile distilled water, and UV-irradiated (254 nm, 15 min each side), then artificially contaminated with SE63 and ST53 (1.0 × 10^8^ CFU/mL). Based on a previous study (Meng et al., 2021), pieces were divided into the CCJY group (50, 25, 12.5 μg/mL), the bacteriophage (CKT1, ФPLL1, ФF118P13 at 8.0 × 10^10^ PFU/mL), the CNCA control group, and the PBS control group. Each piece received 0.125 mL spray treatment and was stored at 4 °C. Samples were collected at 1, 2, 12, 24, 48, and 72 h for total bacterial count determination (Meng et al., 2021). For preservation evaluation, fresh chicken pieces were treated with CCJY at different concentrations or CNCA control by spraying (0.125 mL/piece) and stored at 4 °C. At designated time points, sensory characteristics (Supplementary Table S2), pH, drip loss rate (calculated as (initial mass at sampling)/initial mass×100%), TVB-N content (determined by Kjeldahl analyzer), TBARS value (measured by fluorescence assay kit (Ni et al., 2023)), and H&E-stained tissue sections were evaluated.

### 2.9 Microbiome diversity analysis

Total DNA was extracted from chilled chicken meat samples stored for 0, 4, and 8 days. The V3–V4 hypervariable region of the 16S rRNA gene was amplified using the primers 338F and 806R. Amplicon libraries were constructed and sequenced on the Illumina NovaSeq platform with paired-end (PE250) mode by Personal Biotechnology Co., Ltd. (Shanghai, China). Raw sequencing data were processed using Cutadapt for quality control, followed by analysis in QIIME2 2019.4 with the DADA2 plugin. DADA2 was employed for quality filtering, denoising, read merging, and chimera removal (Callahan et al., 2016). Sequences with 100% similarity were merged to generate amplicon sequence variants (ASVs), without performing traditional OTU clustering. ASVs with an abundance lower than 0.001% of the total sequencing depth across all samples were removed. Taxonomic annotation was performed by aligning ASV representative sequences against the Greengenes database (DeSantis et al., 2006). This approach enabled comprehensive characterization of the microbial community composition and diversity in chilled chicken samples under different storage conditions.

### 2.10 Data analysis

Statistical analyses were performed using GraphPad Prism 8.0. Data are presented as the mean ± standard deviation. Comparisons between two groups were performed using Student's t-test, and comparisons among multiple groups were performed using one-way analysis of variance. The significance level was set at p = 0.05. Statistical significance was denoted as follows: *p* ≥ 0.05 (ns); *p* < 0.05 (∗); *p* < 0.01 (∗∗); *p* < 0.001 (∗∗∗); *p* < 0.0001 (∗∗∗∗). All experiments were independently repeated three times.

## 3 Results and discussion

### 3.1 Synthesis and characterization of CCJY nanoparticles

**Fig. 2:**
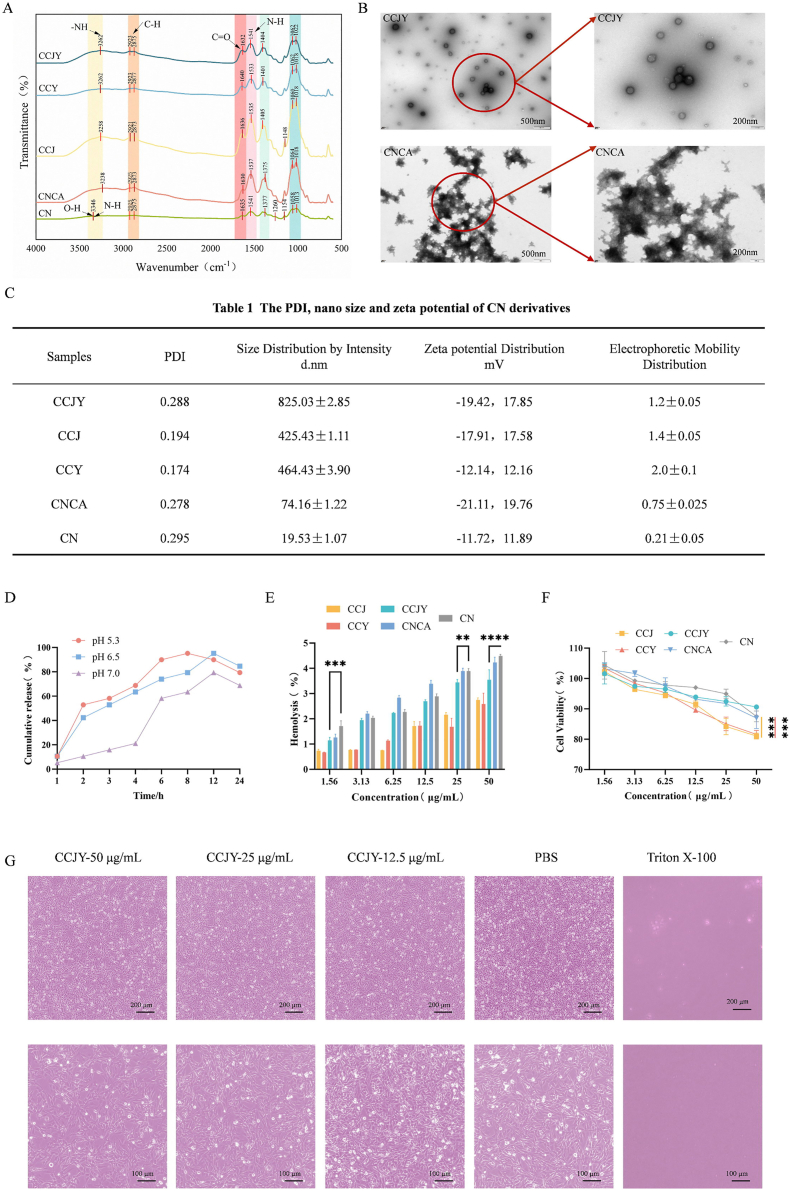
Physicochemical characterization and biocompatibility evaluation of CCJY nanoparticles. (A) FTIR of CCJY and CN derivatives;(B) TEM images of CCJY and CNCA nanoparticles;(C) PDI, nanoparticle size, and Zeta potential table of CCJY and CN derivatives;(D) Durg release of CCJY from PBS buffer under different pH conditions;(E) Hemolytic activity of CCJY and CN derivatives evaluated using red blood cell hemolysis assay;(F) Effect of CCJY and CN derivatives and DF-1 cell viability detected using MTT assay;(G) Comparison of DF-1 cell morphology between different concentration of CCJY treatment and the control groups.

In this study, a chitosan-based nanocomposite, CCJY (CNCA-MccJ25-MccY), was constructed by encapsulating the complementary lasso peptides MccJ25 and MccY within a citric acid-crosslinked chitosan matrix, along with its derivatives CCJ (CNCA-MccJ25), CCY (CNCA-MccY), CNCA, and CN were constructed. Subsequently, their biological activities were characterized, and their application potential in extending the shelf life of meat and egg products was analyzed (Fig. 1). The nanostructures were systematically characterized by FTIR. The results (Fig. 2A) showed that in the spectrum of CN, the band at 3346 cm^−1^ was attributed to O–H and N–H stretching vibrations, while the absorptions at 1635, 1541, and 1377 cm^−1^ corresponded to amide I (C=O stretch), amide II (N–H bend), and amide III (C–N stretch) bands, respectively, consistent with the findings reported by Agarwal, M et al. (Agarwal et al., 2018). For CNCA, CCJ, CCY, and CCJY, the N–H peak at 3345 cm^−1^ broadened and shifted, indicating the formation of secondary amines (–NH–). Shifts and broadening in the C–H stretching vibration region (2875–2925 cm^−1^) confirmed the coexistence of CA, MccJ25, and MccY. Further evidence for structural changes came from the bands at 1640 cm^−1^ (C=O stretch), 1401 cm^−1^ (C–N bend), and 1060 cm^−1^ (C–O stretch). The broadening of the N–H bending vibration peak at 1535 cm^−1^ indicated the consumption of primary amines and formation of secondary amines, suggesting successful crosslinking between CN and MccJ25/MccY via amide bonds to form CCJY. The TEM images (Fig. 2B) further revealed that CNCA exhibited an irregular porous network structure with a rough surface, whereas CCJY displayed a nearly spherical and regular structure. The particle size observed using TEM was approximately 100-200 nm. The hydrodynamic diameters and zeta potentials of the synthesized materials were determined using DLS (Fig. 2C and S1). The results showed (Table 1) that the hydrodynamic diameters of CCJY, CCJ, CCY, CNCA, and CN ranged from 19 to 825 nm, with polydispersity indices (PDI) all below 0.3. Specifically, CCJY exhibited a hydrodynamic diameter of 825.03 ± 2.85 nm, a PDI of 0.288, and a zeta potential of −19.42 ± 17.85 mV. The notable discrepancy between the particle sizes measured by TEM (100-200 nm) and DLS (825 nm) is attributable to their fundamental measurement principles. TEM visualizes the physical core size of nanoparticles in a dehydrated state, whereas DLS determines the hydrodynamic diameter, which includes the hydration layer and surface-bound polymers in an aqueous environment. As previously reported, these methodological differences can lead to size variations of up to 200-300% (Filippov et al., 2023). These fundamental differences in measurement principles account for the systematic variation observed between the two techniques. Collectively, these results confirm the successful construction of the chitosan-lasso peptide composite nanomaterial CCJY, characterized by effective amide bond crosslinking, a regular spherical morphology, good dispersibility, and excellent stability. To investigate the effect of pH on the drug release behavior of CCJY and evaluate its pH-responsive release characteristics, the cumulative release profiles were measured in PBS buffers with different pH values. The results showed (Fig. 2D) that the drug release of CCJY increased in a time-dependent manner under all three pH conditions (pH 5.3, 6.5, and 7.0), with release profiles varying across different pH levels. Specifically, the release rate was the fastest and the cumulative release amount was the highest (95.27%) at pH 5.3. As the pH increased to 6.5 and 7.0, both the release rate and the final release extent decreased progressively. The pH-dependent release behavior of CCJY, characterized by accelerated release under acidic conditions, suggests potential utility in food preservation contexts where localized pH decreases occur (Meng et al., 2024; Liang et al., 2024), and may inform the design of controlled antimicrobial delivery strategies.

**Table 1:** The PDI, nano size and zeta potential of CN derivatives.

Samples	PDI	Size Distribution by Intensity d.nm	Zeta potential Distribution mV	Electrophoretic Mobility Distribution
CCJY	0.288	825.03 ± 2.85	−19.42,17.85	1.2 ± 0.05
CCJ	0.194	425.43 ± 1.11	−17.91,17.58	1.4 ± 0.05
CCY	0.174	464.43 ± 3.90	−12.14,12.16	2.0 ± 0.1
CNCA	0.278	74.16 ± 1.22	−21.11,19.76	0.75 ± 0.025
CN	0.295	19.53 ± 1.07	−11.72,11.89	0.21 ± 0.05

### 3.2 The biocompatibility, excellent stability, and encapsulation efficiency of CCJY

**Fig. 3:**
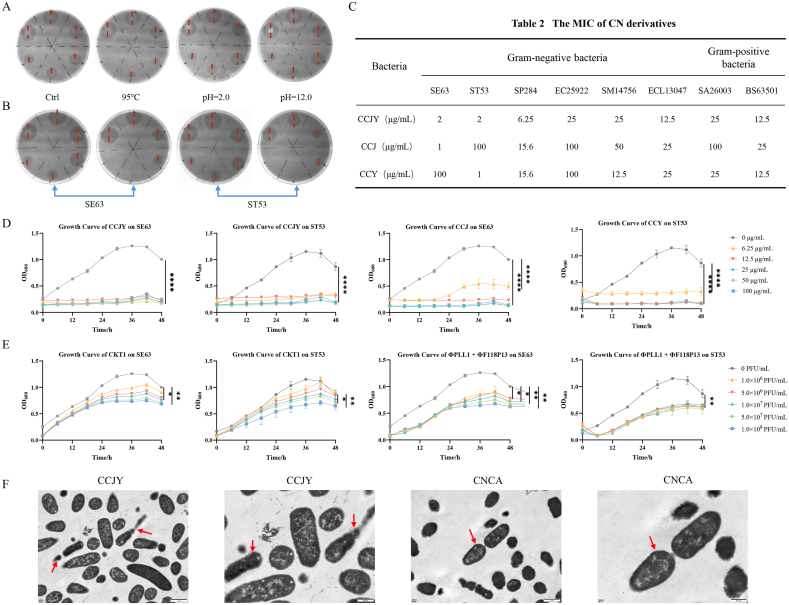
Evaluation of stability, encapsulation efficiency, and antibacterial activity of CCJY. (A)Antibacterial activity of CCJY under different temperature and pH conditions;(B) Evaluation of antibacterial activity of CCJY dialysis internal and external solutions against SE63 and ST53;(C) MIC values of CCJY, CCJ, and CCY against bacteria;(D) Groth inhibition curves of CCJY, CCJ, and CCY against SE63 and ST53; (E)Growth inhibition curves of CKT11, ΦPLL1, and ΦF118P13 phages against SE63 and ST53;(F) Effect of drug treatment on the ultrastructure of SE63. A Scanning electron microscopy study at 500 nm and 1 μm scales.

The biocompatibility of the CCJY nanomaterials was evaluated using hemolysis and cytotoxicity assays. Hemolysis assay results (Fig. 2E) showed that at 25 μg/mL, the hemolysis rate of CCJY was 3.44% ± 0.13%, remaining below the 5% safety threshold and comparable to that of the negative control CN (3.89% ± 0.1%). These results indicate that CCJY exhibits favorable hemocompatibility within the tested concentration range. When the concentration increased to 50 μg/mL, the hemolysis rate of CCJY was 3.54% ± 0.4%, still below the 5% safety threshold. According to Nájera's research, materials with hemolysis rates below 5% are considered non-hemolytic and safe for biomedical and food contact applications (Nájera-Maldonado et al., 2025). The effect on DF-1 cell metabolic activity was assessed using the MTT assay, combined with cell morphology observation and hemolysis tests. Across a concentration gradient of 1.56–50 μg/mL (Fig. 2F), the viability of DF-1 cells showed a slow declining trend as the concentration of CCJY and its derivatives increased. In the low-concentration range (1.56–6.25 μg/mL), cell viability remained between 95% and 100%, showing no significant difference from the negative control. At higher concentrations (12.5–50 μg/mL), cell viability decreased slightly in a concentration-dependent manner but remained above 80% and was significantly better than the positive control. According to Burkhardt et al. (2022), a reduction in cell viability by more than 30% is considered cytotoxic. Cell morphology observations (Fig. 2G) showed that DF-1 cells treated with different concentrations of CCJY maintained a typical fibroblast morphology, with clear cell boundaries, well-extended cytoplasm, and characteristics of tight arrangement and normal spreading. Compared with the untreated control group, no toxicity-related morphological changes, such as cell shrinkage, membrane integrity disruption, or detachment, were observed in any of the treatment groups. The stability and encapsulation performance of CCJY were evaluated using thermal/acid/alkali stability tests and dialysis-based encapsulation efficiency measurements. Spots on law assay results (Fig. 3A) showed that the antimicrobial activity of CCJY and its derivatives did not change significantly after treatment at 95 °C for 2 h or under pH 2.0 or 12.0 conditions for 2 h, compared with the room temperature control. Furthermore, the dialysis-spot method (Fig. 3B) revealed that the encapsulation efficiency of CCJY exceeded 96.67%.This exceptionally high encapsulation efficiency is attributable to the citric acid-mediated covalent crosslinking between chitosan and the lasso peptides, which forms stable amide bonds that minimize peptide leakage during nanoparticle formation (Jiang et al., 2023). CCJY nanomaterials (CA-crosslinked, encapsulation efficiency>96.67%) show the stability under 95 °C and pH 2.0/12.0, with cytocompatibility and hemocompatibility.

### 3.3 The potential of antibacterial activity against gram-positive and gram-negative pathogens of CCJY

The *in vitro* antibacterial activity of CCJY was evaluated by determining the MIC against various foodborne pathogens using a microbroth dilution method. Based on the MIC results (Fig. 3C–Table 2), CCJY exhibited significant inhibitory activity against both gram-negative and gram-positive bacteria. The MIC values for *S. serovars* (ST53, SE63, SP284) ranged from 2 to 6.25 μg/mL. The MIC was 25 μg/mL for *E. coli* and *Serratia* Marcescens, and 12.5, 25, and 12.5 μg/mL for *Enterobacter* Cloacae, *Staphylococcus* Aureus, and *Bacillus* Subtilis, respectively. In contrast, CCJ and CCY showed narrow antibacterial spectra. To clarify the interaction type between CCJ and CCY in combination, this study evaluated their combined antibacterial effects against multiple bacterial strains using the fractional inhibitory concentration index (FICI). The results (Table S3) indicated that the combined effect was highly species-specific: a synergistic effect was observed against *Salmonella* Pullorum (SP284), *E. coli* (EC25922), and *Enterobacter* Cloacae (ECL13047); an additive effect was observed against *Salmonella* Enteritidis (SE63), *Salmonella* Typhimurium (ST53), and *Serratia* Marcescens (SM14756); whereas an indifferent effect was observed against *S. aureus* (SA26003) and *B. subtilis* (BS63501). The broad-spectrum activity of CCJY, including its notable effect against Gram-positive bacteria like *S. aureus* and *B. subtilis*, stems from the coordinated function of its three active constituents within the nanocarrier. Chitosan mediates initial bacterial attachment via electrostatic forces (Kuang et al., 2023), MccJ25 targets Gram-negative bacteria through inhibition of RNA polymerase (Yu et al., 2018). MccY exhibits activity against Gram-positive strains including *S. aureus* and *B. subtilis* (Li et al., 2021). The nanoparticle formulation may facilitate accumulation of these peptides at the bacterial surface, where chitosan-mediated attachment and the complementary activities of MccJ25 and MccY collectively contribute to the observed bactericidal activity against both Gram-negative and Gram-positive bacteria.

**Table 2:** The MIC of CN derivatives.

Bacteria	Gram-negative bacteria						Gram-positive bacteria	
	SE63	ST53	SP284	EC25922	SM14756	ECL13047	SA26003	BS63501
CCJY(μg/mL)	2	2	6.25	25	25	12.5	25	12.5
CCJ(μg/mL)	1	100	15.6	100	50	25	100	25
CCY(μg/mL)	100	1	15.6	100	12.5	25	25	12.5

To further evaluate the sustained antibacterial effect, growth curves were analyzed for SE63 and ST53 strains treated with different concentrations (6.25–100 μg/mL) of CCJY. The results (Fig. 3D) showed that the OD values of the CCJY-treated strains did not increase significantly over 72 h, indicating sustained growth inhibition, whereas those of the PBS control group exhibited a typical normal growth trend. Regarding specificity, CCJ showed activity only against SE63, and CCY only against ST53, whereas CCJY significantly inhibited both test strains, confirming its broad-spectrum antibacterial activity. As shown in Fig. 3E, treatment with *Salmonella* phages (CKT1, ФPLL1, and ФF118P13) prolonged the bacterial lag phase but did not achieve complete inhibition. This limited efficacy may be attributed to the receptor-dependent mechanism of these phages, which restricts their host range to specific serovars and renders them susceptible to resistance through single-point mutations in bacterial surface receptors (Tung et al., 2025). In contrast, CCJY integrates chitosan-mediated electrostatic interactions with the combined activities of MccJ25 and MccY. The nanoparticle formulation may promote localized peptide enrichment at bacterial surfaces, and this multi-target strategy may account for its observed antibacterial spectrum while potentially lowering the risk of resistance emergence. Scanning electron microscopy (SEM) observations revealed that *Salmonella* cells in the untreated control group exhibited intact morphology, smooth surfaces, clear boundaries, and typical short rod shapes (Fig. 3F). In contrast, *Salmonella* treated with CCJY showed significant cell membrane damage, characterized by noticeable shrinkage, depression, and pore formation on the cell surface. Some bacterial cells were deformed, ruptured, or even leaked cytoplasmic contents. These morphological alterations demonstrate that CCJY significantly disrupts the integrity of the *Salmonella* cell membrane, consistent with its proposed multi-target membrane disruption mechanism.

### 3.4 The effects of CCJY on antibacterial properties applied in egg and chilled chicken surfaces

**Fig. 4:**
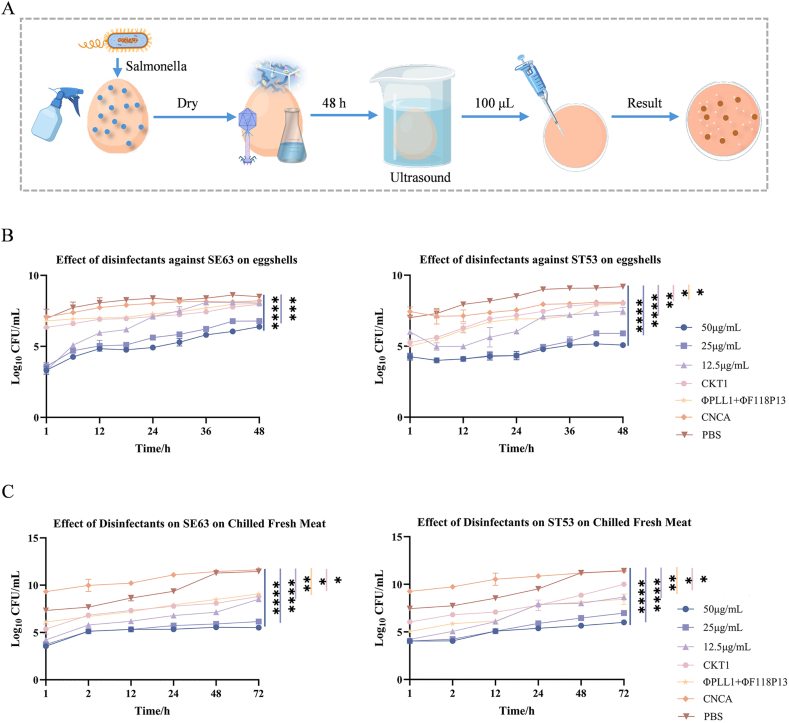
Evaluation of the antibacterial effect of CCJY against *Salmonella* on the surface of eggs and chicken meat. (A) Schematic diagram of sterilization process *Salmonella* on the surface of eggs; (B) Changes in colony counts onthe surface of eggs in different treatment groups; (C) Changes in colony counts on the surface of chicken meat in different treatment groups.

To assess the efficacy of CCJY against *Salmonella* on egg surfaces, the antibacterial activities of different concentrations were compared over 48 h (Fig. 4A). Fig. 4B showed that the bacterial counts in CCJY treatment groups (50, 25, and 12.5 μg/mL) increased slowly over time. The high-concentration groups (50 and 25 μg/mL) had significantly lower bacterial counts at all time points compared with the low-concentration group (12.5 μg/mL). Specifically, at 48 h, the bacterial count for the 50 μg/mL CCJY group was 6.39 ± 0.09 log_10_ CFU/mL, significantly lower than that of the 12.5 μg/mL group (8.05 ± 0.02 log_10_ CFU/mL, *p* < 0.05). No significant difference was observed between the 25 μg/mL group (6.79 ± 0.05 log_10_ CFU/mL) and the 50 μg/mL group. The antibacterial effect of the 12.5 μg/mL group was limited and not significantly different from the PBS control group (8.51 ± 0.04 log_10_ CFU/mL). However, the bacterial counts in the phage treatment group were consistently lower than those in the control but higher than those in the CCJY treatment groups, suggesting that CCJY at appropriate concentrations possesses superior antibacterial potential compared with the phages. Similarly, in the chilled chicken surface experiment (Fig. 4C), the bacterial counts in the high-concentration CCJY groups (50 and 25 μg/mL) were significantly lower than those in the 12.5 μg/mL group at all time points. The 25 μg/mL group showed a significant reduction of 2.37 log_10_ CFU/g compared with the 12.5 μg/mL group at 72 h. In contrast, the PBS control and CNCA groups maintained high bacterial counts (11.6 and 11.0 log_10_ CFU/g at 72 h, respectively), both significantly higher than the CCJY treatment groups. These results indicate that CCJY also has significant antibacterial effects on egg and chilled chicken surfaces, and 25 μg/mL is the optimal concentration balancing antibacterial efficiency and concentration dependency. The observed 2.37 log_10_ CFU/g reduction on chicken meat is greater than the 1.4–1.5 log_10_ CFU/cm^2^ reduction documented for *Salmonella* phages in previous studies (Kim et al., 2024). CCJY also exhibited 99.9% bactericidal efficacy against *Salmonella* on egg and chicken surfaces, attributed to the formation of a uniform antimicrobial nanocoating that enabled sustained peptide release.

### 3.5 CCJY effectively maintains egg storage quality and extends shelf life

**Fig. 5:**
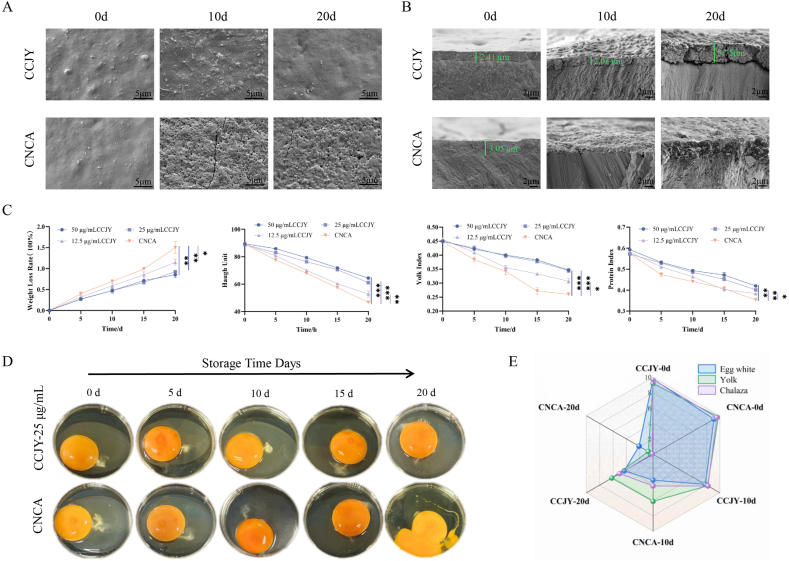
Evaluation of the preservation effect of CCJY on eggs. (A) SEM images of the effect of CCJY treatment on the microstructure of the eggshell cross section;(B) SEM images of the effect of the CCJY treatment on the microstructures of the eggshell cross section; (C) Effects of different concentration of CCJY treatment on quality indicators of eggs during storage, including weight loss rate, Haugh unit, yolk index, and albumen index;(D) Apparent comparison of eggs between the CCJY treatment group and the CNCA control group during storage; (E) Radar chart of the comprehensive evaluation of egg quality under CCJY treatment.

The effect of CCJY nanoparticles on egg storage quality was investigated by assessing both apparent and internal quality changes. And the influence of different CCJY concentrations and the CNCA control group on egg storage quality indicators was evaluated (Table S4). SEM analysis clearly revealed microstructural differences between the 25 μg/mL CCJY treatment group and the CNCA control group at 0, 10, and 20 days. Surface structural observations (Fig. 5A) showed that the eggshells in the CCJY group remained relatively smooth and intact throughout storage, with clear and uniformly distributed pore structures. In contrast, the CNCA control group exhibited increased porosity, enlarged pores, and increased surface roughness over time, displaying typical characteristics of calcium carbonate dissolution and organic matrix degradation. From the cross-sectional structure (Fig. 5B), eggshell thickness in the CCJY group showed a slow decreasing trend from day 0 to day 10, which was potentially related to material reorganization caused by water evaporation and CO_2_ escape, followed by a slight increase on day 20. Conversely, the cross-sectional structure of the CNCA control eggshells became progressively looser and more porous, deteriorating severely by day 20 and preventing effective thickness measurement, indicating a significant loss of microstructural integrity. Eggshell quality plays a critical role in determining egg freshness and the risk of microbial contamination during storage (Caner and Yüceer, 2015). Chitosan-based coatings preserve eggshell integrity through moisture barrier formation; however, the influence of antimicrobial peptide incorporation on eggshell microstructure has remained unclear. SEM analysis demonstrated that CCJY treatment maintained eggshell structural integrity throughout the 20-day storage period, whereas CNCA controls exhibited progressive deterioration with increased porosity and surface roughness. This protective effect likely results from the stable crosslinked network formed by chitosan and lasso peptides, which provides enhanced barrier properties relative to the CNCA control.

The results in Fig. 5C showed that, regarding weight loss, both the 50 and 25 μg/mL groups exhibited significantly lower values from day 5 to day 20 than the CNCA control (*p* < 0.01). The final weight loss rates were 0.90% and 0.82%, respectively, with no significant difference between the two groups (*p* > 0.05), but both were significantly lower than the CNCA control group's 1.42% (*p* < 0.01). Unlike quaternary ammonium salt-polymer coatings, which reduce egg weight loss solely through physical pore blocking (Almeida e Silva et al., 2020), CCJY integrates barrier formation with active antibacterial activity. This dual functionality enabled CCJY to achieve a comparably low weight loss rate (0.82%) while significantly reducing bacterial loads on eggshell surfaces (Fig. 4B). The HU analysis showed that CCJY treatment effectively delayed this decline. The 25 μg/mL group maintained a relatively high level of 61.12 at the end of storage, indicating better maintenance of protein viscosity and freshness. For the protein index, the values decreased from 0.598 (day 5) to 0.437 (day 20) in the 50 μg/mL group, and from 0.581 to 0.431 in the 25 μg/mL group, both significantly higher than the CNCA control group (*p* < 0.01), suggesting that CCJY effectively delayed the liquefaction of thick albumin. The YI changes further supported this conclusion; the final values for the 50 and 25 μg/mL groups were 0.358 and 0.352, respectively, significantly higher than the CNCA control group's 0.255 (*p* < 0.001), indicating that CCJY treatment significantly inhibited yolk membrane weakening and flattening. Apparent structure observation revealed that in the 25 μg/mL CCJY group, the reduction rate of thick albumin slowed down, still distinguishable from thin albumin at day 20, with the chalazae structure largely intact and the yolk morphology complete (Fig. 5D). In the CNCA control group, the thick albumin almost completely disappeared by day 10, the chalazae detached by day 15, and the yolk broke by day 20. As shown in the sensory evaluation radar chart (Fig. 5E), CCJY treatment led to improved quality attributes of the yolk, albumen, and chalazae compared with the CNCA control. Thus, 25 μg/mL CCJY effectively preserves egg storage quality: it reduces weight loss, maintains high Haugh unit and protein viscosity, stabilizes albumin and yolk, preserves intact chalazae and yolk morphology, and sustains eggshell integrity compared to the CNCA control's deteriorated, porous shell. Notably, the sensory evaluation was performed by a small panel of only three trained assessors without official certification, and no consumer hedonic validation was conducted. Future studies with larger assessor panels and consumer sensory testing are needed to further verify the present results.

### 3.6 CCJY significantly improves chilled chicken storage quality and inhibits spoilage progression

**Fig. 6:**
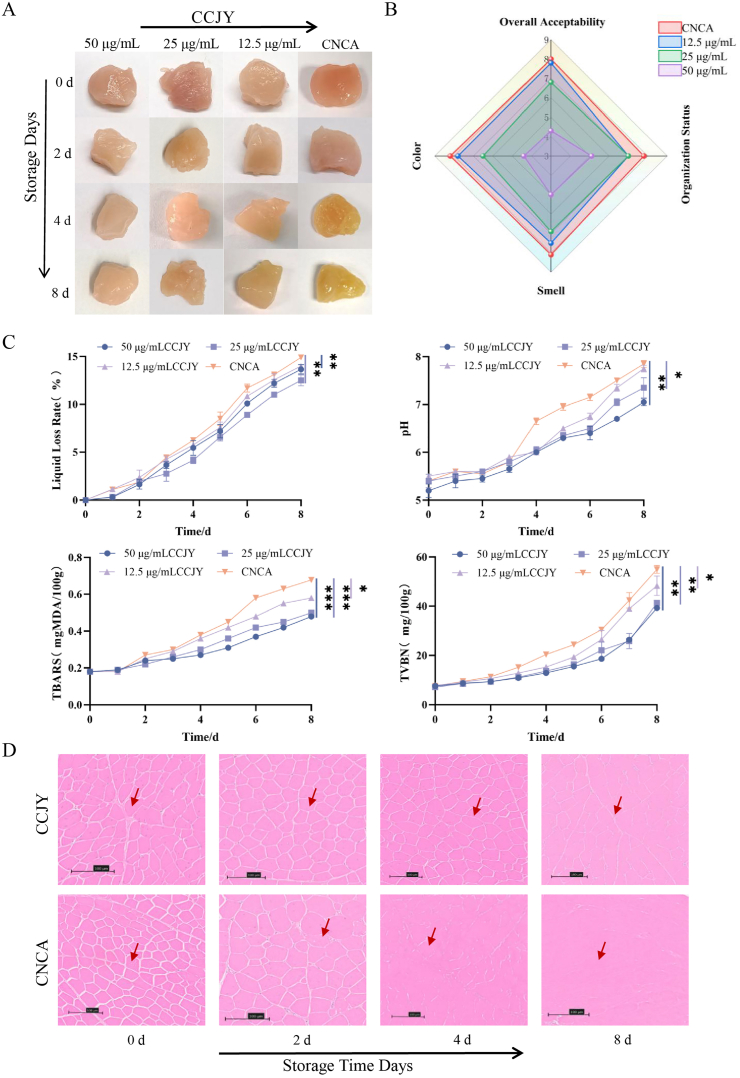
Evaluation of the preservation effect of CCJY on chilled meat. (A) Apparent comparison of chicken meat between the CCJY treatment and the CNCA control groups during storage;(B) Radar chart of the effect of CCJY treatment on chicken meat quality on the 4th day of storage;(C) Effects of different concentration of CCJY treatment on quality indicators of chicken meat during storage, including drip loss rate,pH, TVBN;(D)H&E-stained section of the effect of CCJY treatment on the microstructure of chicken tissue.

The preservative effect of CCJY nanoparticles on chilled chicken was evaluated by systematically measuring sensory and physicochemical indicators during storage. Multiple indicators demonstrated that CCJY treatment groups, particularly at 25 μg/mL, exhibited significant preservative effects. Sensorily (Fig. 6A and B), the 50 and 25 μg/mL groups maintained uniform color and slight meat odor even at day 8, without obvious putrid sour odor or sticky surface, whereas the CNCA control group showed significant browning, rancidity, and texture deterioration. Physiochemically, CCJY treatment effectively delayed the deterioration of various quality indicators (Fig. 6C and Table S5). The drip loss rate in the 25 μg/mL group was 12.5 %, significantly lower than the 14.9 % in the CNCA control group (*p* < 0.001). Regarding pH changes, CCJY treatment delayed the rise in meat pH during storage. On day 8, the pH value of the 25 μg/mL group was 7.35, significantly lower than that of the CNCA control group (7.85, *p* < 0.01), indicating that CCJY effectively suppressed the accumulation of alkaline metabolites from microbial spoilage, likely through inhibition of specific spoilage bacteria such as *Pseudomonas* and *Acinetobacter*, whose metabolic activity elevates pH during protein decomposition. The TBARS value in the CCJY group (0.54 mg MDA/100g at day 8) was significantly lower than in the CNCA control (0.68 mg MDA/100g), indicating that CCJY effectively inhibited lipid oxidation in chilled chicken. The TBARS results are consistent with the trends reported by Meng (Meng et al., 2021). Compared to the direct application of free antimicrobial peptides, CCJY treatment extended the shelf life of chilled chicken by 3-4 days under the same conditions. In H&E-stained sections of chilled chicken treated with 25 μg/mL CCJY (Fig. 6D), tightly arranged cells with clearly visible nuclei were observed at day 0; mild nuclear pyknosis but intact structure was observed at day 4; and looser cells with blurred nuclear edges, yet not completely disappeared, were noted at day 8. In the CNCA control group, cells were also tightly arranged at day 0, similar to the treatment group; cell gaps began to disappear and nuclei started to dissolve, with some areas showing complete nuclear loss, at day 4; and by day 8, cell gaps disappeared, nuclei were completely dissolved, and the tissue became loose. CCJY could significantly delay the sensory and physicochemical deterioration of chilled chicken, and 25 μg/mL is identified as its optimal preservative concentration.

### 3.7 CCJY treatment significantly alters the microbial community structure in chilled chicken during storage

**Fig. 7:**
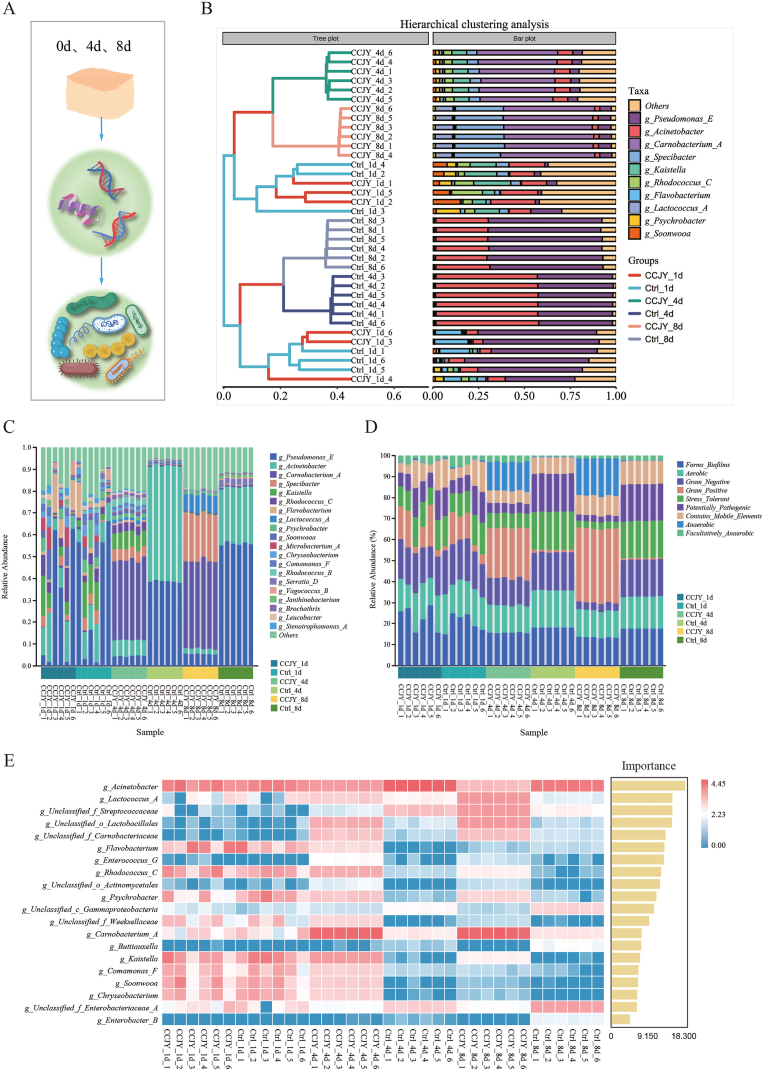
Effect of CCJY on the microbial community structure of chilled chicken meat. (A)Schematic diagram of the analysis of the effect of CCJY treatment on the chicken meat microbial community based on 16S rRNA sequencing;(B) Hierarchical clustering analysis;(C) Species composition analysis;(D) BugBase analysis;(E) Random forest analysis.

To investigate the impact of CCJY treatment on the microbial ecology of chilled chicken during storage, a multi-dimensional analysis was conducted on samples treated with 25 μg/mL CCJY and collected on days 0, 4, and 8 (Fig. 7A). The experimental results demonstrated high intragroup reproducibility and data reliability. As shown in Fig. 7B, the microbial community structure in the CCJY-treated group remained relatively stable throughout the storage period, whereas that in the CNCA control group exhibited significant succession, ultimately forming a terminal spoilage community (Fig. S2 and S3, Tables S6 and S7). In terms of species composition, CCJY treatment effectively suppressed the proliferation of key spoilage-related bacteria, such as *Pseudomonas* and *Acinetobacter*, which dominated the control group by the end of storage (Fig. 7C). Functionally, BugBase analysis revealed that CCJY promoted a phenotypic shift from a spoilage-prone to a more stable community type (Fig. 7D). Concomitantly, competitive flora including *Carnobacterium* and *Lactococcus*, which are associated with slower spoilage progression, were enriched in the treated samples (Fig. 7E–Table S8) (Lauritsen et al., 2019). Linear discriminant analysis effect size analysis further identified differential biomarkers between the groups, with the CNCA control group enriched in spoilage-related bacteria and the CCJY group enriched in beneficial lactic acid bacteria (Figs. S4 and S5). Analysis of the community assembly mechanism indicated that this process was primarily deterministic (Fig. S6, Table S9), confirming that the CCJY intervention effectively modulated the microbiota in a directed manner. Therefore, CCJY treatment modulates the microbial community structure of chilled chicken by specifically suppressing the competitive advantage of major spoilage bacteria such as *Pseudomonas* and *Acinetobacter*. By inhibiting these key taxa, meat spoilage is effectively delayed, thereby presenting a reliable strategy for shelf-life extension of chilled chicken. Collectively, these findings demonstrate that CCJY nanoparticles possess broad-spectrum antimicrobial activity, excellent biocompatibility, and significant potential to extend the shelf life of eggs and chilled chicken by modulating spoilage microorganisms and preserving food quality. It is important to note, however, that these results were obtained under controlled laboratory conditions. Therefore, the translational application of CCJY as a food preservative would benefit from further investigation. Future efforts may include validating its efficacy and stability under industrial production and supply chain conditions, as well as conducting comprehensive *in vivo* oral safety studies to assess its suitability for human consumption.

## 4 Conclusion

In this study, novel chitosan-lasso peptide composite nanoparticles (CCJY) were successfully developed and showed good biocompatibility and stability under extreme conditions. Compared with chemical disinfectants and bacteriophages, CCJY presents an alternative by avoiding chemical residues and potentially reducing resistance risks due to its multi-target mechanism. CCJY exerted broad-spectrum antimicrobial activity (MIC: 2-25 μg/mL) and suppressed key spoilage bacteria (*Pseudomonas* and *Acinetobacter*), thereby reshaping the microbial community and delaying spoilage. On food surfaces, CCJY achieved 99.9% bactericidal efficacy against *Salmonella*. In preservation assays, 25 μg/mL CCJY reduced bacterial counts and drip loss, maintained egg and chicken quality, and extended shelf life by 3-4 days, outperforming the CNCA control group.

## Funding

This study was supported by Key Laboratory for prevention and control of Avian Influenza and Other Major Poultry Diseases, Ministry of Agriculture and Rural Affairs, PR. China/Guangdong Province Key Laboratory of Livestock Disease Prevention, (Grant No. YDWS202409); Young Scientists of Fund (Type C) of National Natural Science Foundation of China （NSFC）, (Grant No. 32402893); Postdoctoral Fellowship Program of CPSF, (Grant No. GZC20230854); Guangdong Poultry Industry Technology System (2024CXTD20); Guangdong Poultry Industry Technical System Disease control post expert (2023KJ128); National Broiler Industry Technical System (CARS-41); Rural Revitalization Strategy of Guangdong Province (5500-F23015).

## Declaration of competing interest

This study has no interest conflicts. This study was supported by Key Laboratory for prevention and control of Avian Influenza and Other Major Poultry Diseases, Ministry of Agriculture and Rural Affairs, PR. China/Guangdong Province Key Laboratory of Livestock Disease Prevention Open Project, (Grant No. VDWS202409); National Natural Science Foundation of China, (Grant No. 32402893),Expert on in Project Funding. Postdoctoral Fellowship Program of CPSF, (Grant No. GZC20230854); Guangdong Poultry Industry Technology System (2024CXTD20); Guangdong Poultry Industry Technical System Disease control post expert (2023KJ128); National Broiler Industry Technical System (CARS-41); Rural Revitalization Strategy of Guangdong Province (5500- F23015).

## Data Availability

The authors confirm that the data supporting the findings of this study are available within the article and its supplementary information and addition file 1. All data on microbial composition generated and analyzed during the current study is available in NCBI and the accessed number is PRJNA911349, and with this paper into the library to provide reference.
