# Supplementary material for: A chitosan-lasso peptides nanoparticle for enhanced antibacterial activity and fresh-keeping efficacy in eggs and chilled chicken
Source: Curr Res Food Sci. 2026 May 25;12:101448. doi: 10.1016/j.crfs.2026.101448 (PMC13251780; doi:10.1016/j.crfs.2026.101448)
Supplement: Multimedia component 1 [file mmc1.docx]

**Supplementary information**

**A Novel Chitosan-Lasso Peptide Nanoparticle for**

**Enhanced Antibacterial Activity and Fresh-Keeping Efficacy**

**in Eggs and Chilled Chicken**

**Figure S1. Characterization Data of CN Derivatives: Size Distribution and Zeta Potential.**

**Figure S2. PCoA of Microbial Communities in CCJY-Treated Meat.**

**Figure S3. Alpha Diversity of Microbial Communities in CCJY-Treated Meat.**

**Figure S4. NCM Analysis of Microbial Community Assembly.**

**Figure S5. NST Analysis of Microbial Community Assembly.**

**Figure S6. LEfSe Identifies Biomarkers in CCJY-Treated Meat.**

**Table S1 . Sensory evaluation criteria for albumen, chalazae, and yolk qualities of chicken eggs.**

**Table S2 . Sensory evaluation criteria for color, odor, texture, and overall acceptability of chilled chicken during refrigerated storage.**

**Table S3. Data of the fractional inhibitory concentration index (FICI) for CCJ and CCY.**

**Table S4. Data of Egg Freshness Preservation Assessment.**

**Table S5. Data of the Evaluation of Different CCJY Concentrations on the Preservation Effect of Chilled Meat.**

**Table S6-S9 were offered in Additional** **file 1.**

**Table S6. Data of the Alpha Diversity Index in Chilled Fresh Meat Treated with CCJY .**

**Table S7. Data of PCoA on CCJY-Treated Chilled Meat.**

**Table S8. Data of the relative abundance and species composition in CCJY-treated fresh meat.**

**Table S9. Data of LEfSe Analysis on CCJY-Treated Chilled Fresh Meat.**

**Supplementary**


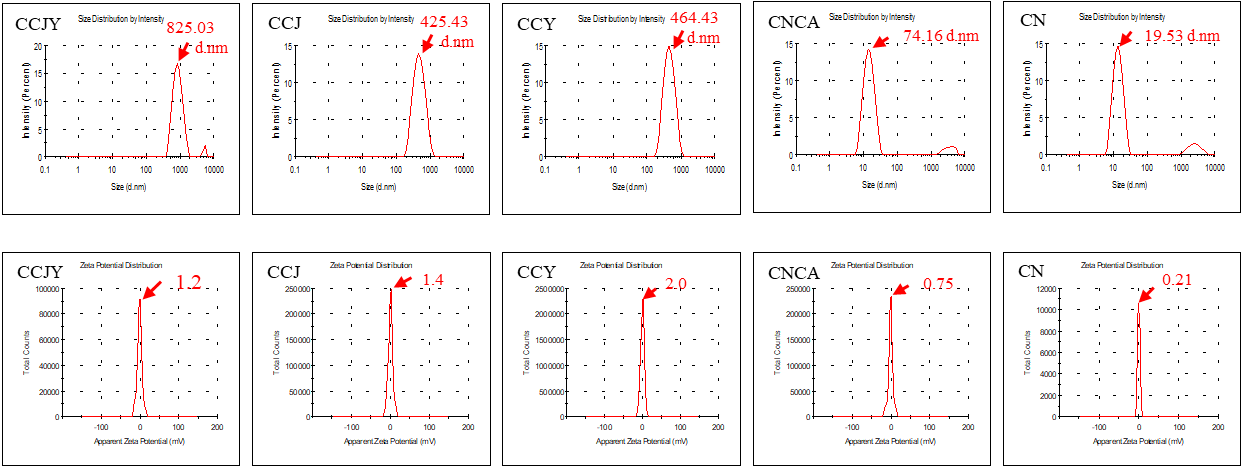
**Figure S1. Characterization Data of CN Derivatives: Size Distribution and Zeta Potential.**

**Figure S2.** **PCoA of Microbial Communities in CCJY-Treated Meat.**

**
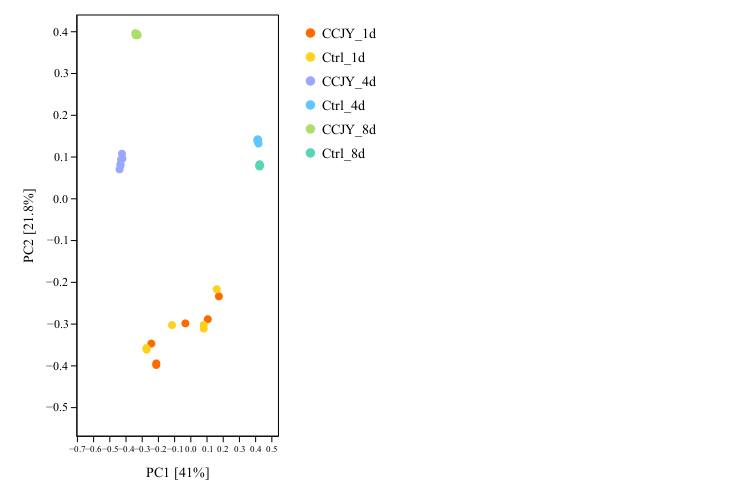
**

**Figure S3. Alpha Diversity of Microbial Communities in CCJY-Treated Meat.**

**
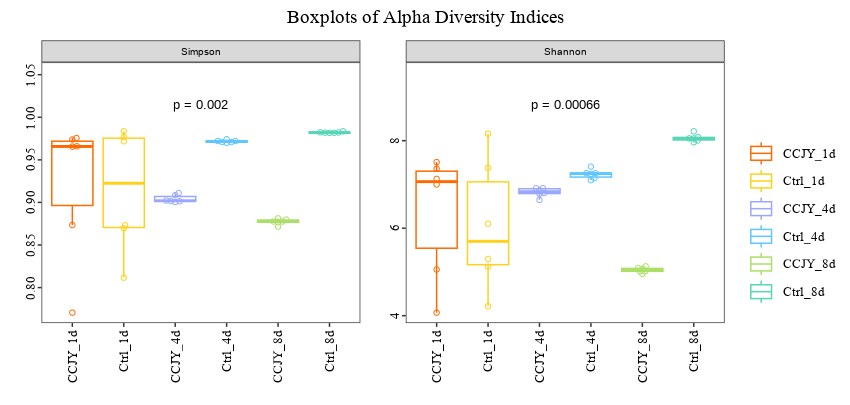
**

**Figure S4. NCM Analysis of Microbial Community Assembly.**

**
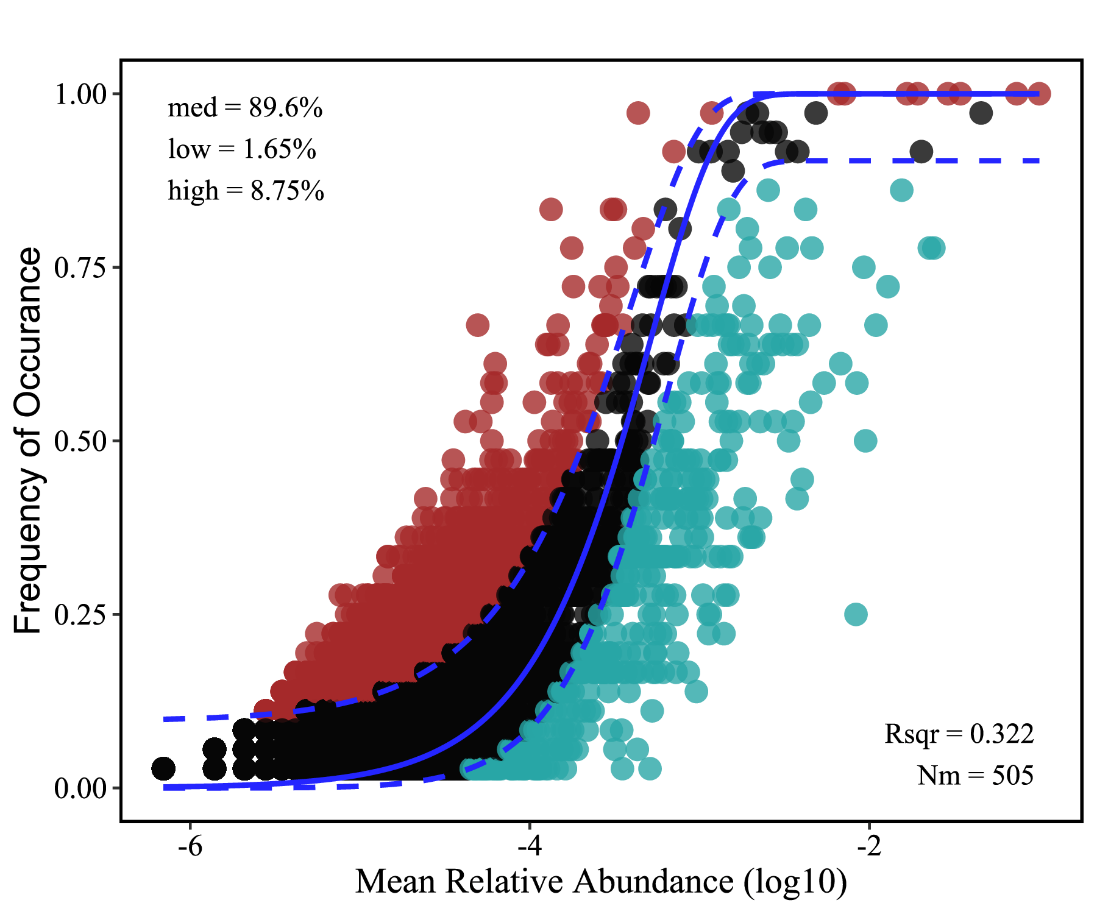
**

**Figure S5.** **NST Analysis of Microbial Community Assembly.**

**
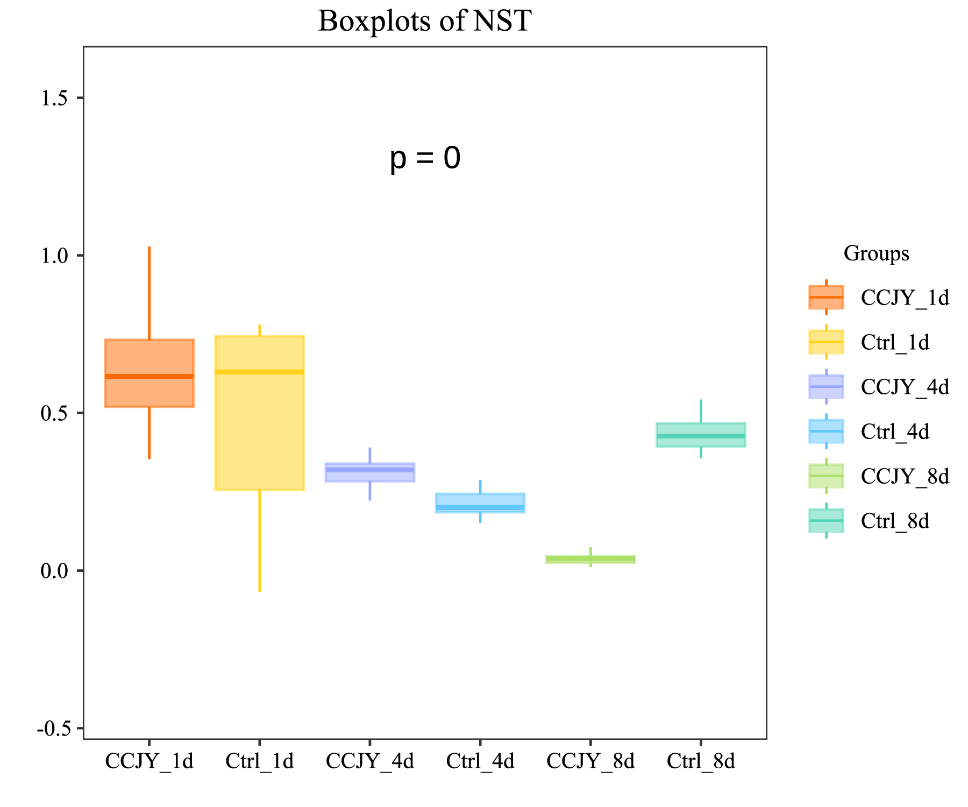
**

**Figure S6. LEfSe Identifies Biomarkers in CCJY-Treated Meat.**

**
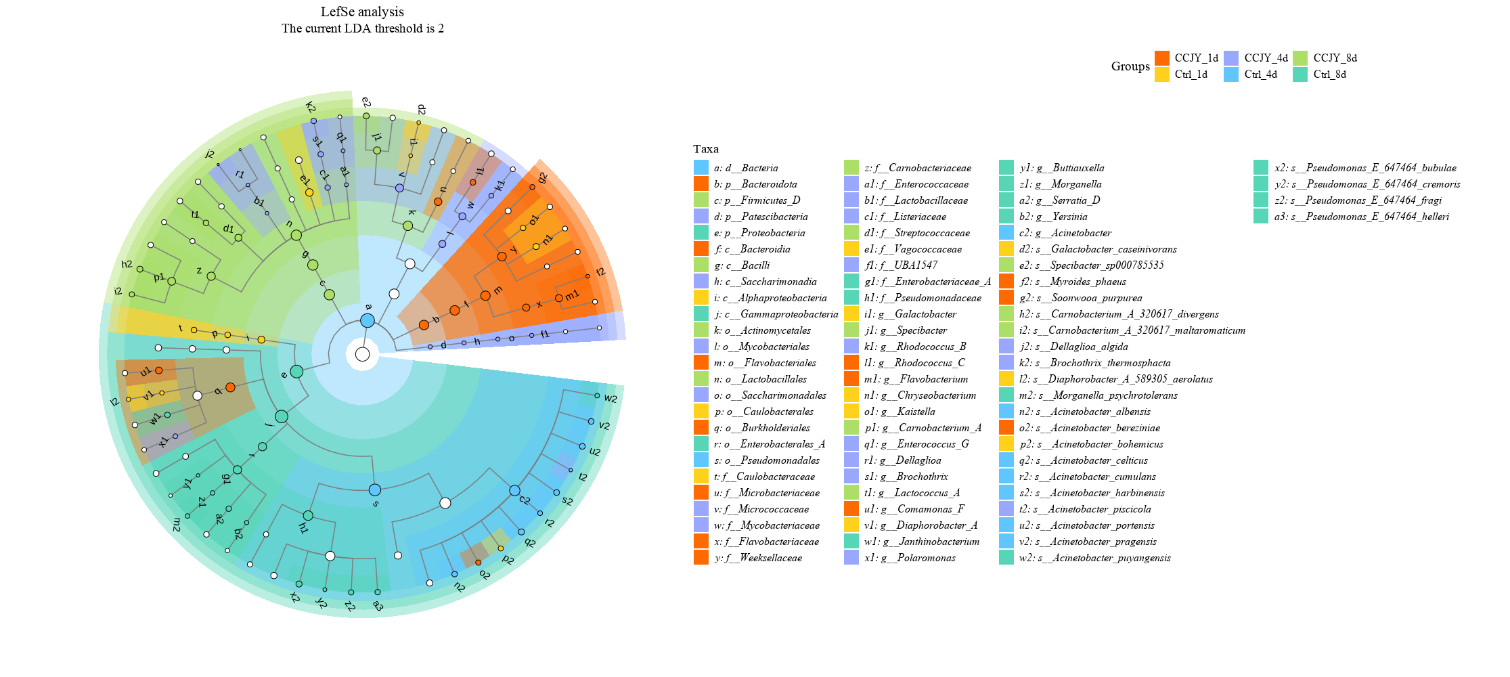

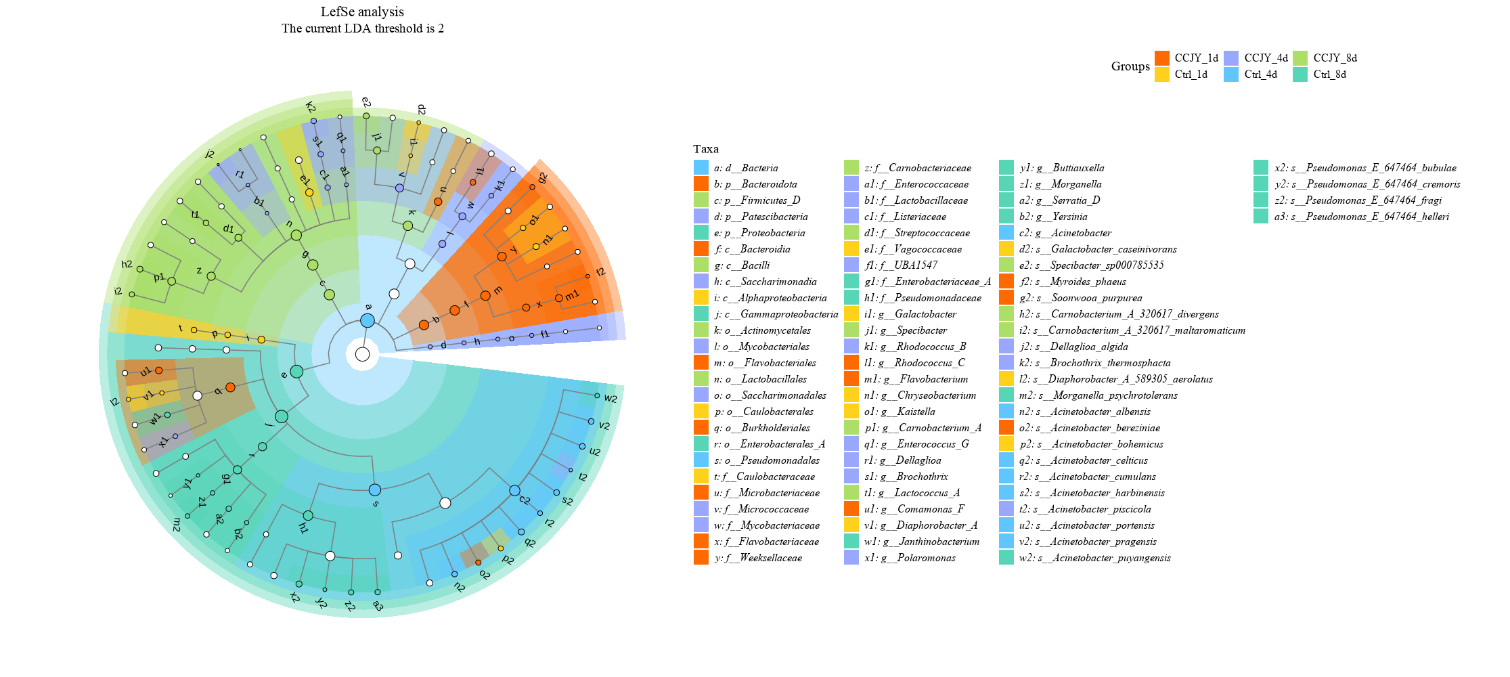
**

| **Score** | **Albumen** | **Chalazae** | **Yolk** |
| --- | --- | --- | --- |
| +++ | Thick albumen abundant, firm/viscous, and no spreading | **Chalazae thick, white/prominent, and intact** | Yolk prominently domed/convex, intact, and resilient (high turgor) |
| ++ | Thick albumen moderately abundant, relatively firm; limited spreading | Chalazae slightly thinned, partially detached/loosened | Yolk slightly flattened, reduced resilience (moderate turgor) |
| + | Thin/spread albumen; thick albumen significantly reduced | Chalazae very thin, tenuous, or completely detached/absent | Yolk flattened, flattened shape |
| - | Thick albumen nearly or completely absent (predominantly thin albumen) | Chalazae absent | Flattened and spread |

**Table S1 .** **Sensory evaluation criteria for albumen, chalazae, and yolk qualities of chicken eggs**

**Sensory evaluation table for chilled chicken**

**This data analysis refers to the references ^1^.**

**Table S2 . Sensory evaluation criteria for color, odor, texture, and overall acceptability of chilled chicken during refrigerated storage.**

**Sensory evaluation table for chilled chicken**

**This data analysis refers to the references^2.^**

| **Parameter** | **Score** | | | |
| --- | --- | --- | --- | --- |
|  | **8-10 (High freshness)** | **6-7.9 (Moderate freshness)** | **3-5.9 (Slight spoilage)** | **0-2.9 (Spoiled)** |
| Color | Bright red | Slightly red (brown) | Dark red (brown) | Grayish white |
| Odor | Characteristic, pleasant | Acceptable | Unpleasant | Putrid, offensive |
| Texture | Firm, elastic, non-sticky | Slightly soft, slightly moist | Soft, exudative | Sticky, excessive exudate |
| Overall acceptability | Excellent | Acceptable | Slightly unacceptable | Unacceptable |

**Table S3. Data of the fractional inhibitory concentration index (FICI) for CCJ and CCY.**

| **Bacterial** | **MIC(CCJ)μg/mL** | **MIC(CCY)**  **μg/mL** | **MIC(CCJ/CCY)μg/mL** | **FICI** | **Action Effect** |
| --- | --- | --- | --- | --- | --- |
| **SE63** | 12.5 | 100 | 6.25/50 | 1 | Addition |
| **ST53** | 25 | 6.25 | 12.5/3.13 | 1 | Addition |
| **SP284** | 25 | 50 | 6.25/6.25 | 0.37 | Synergistic |
| **EC14756** | 125 | 62.5 | 12.5/12.5 | 0.3 | Synergistic |
| **SM14756** | 50 | 25 | 25/12.5 | 1 | Addition |
| **ECL13047** | 25 | 12.5 | 6.25/1.56 | 0.37 | Synergistic |
| **SA26003** | 50 | 25 | 25/25 | 1.5 | Irrelevant |
| **BS63501** | 25 | 12.5 | 12.5/12.5 | 1.5 | Irrelevant |

**FICI value results for CCJ and CCY**

**Table S4. Data of Egg Freshness Preservation Assessment.**

**Weight Loss**

| Days | 50μg/mL | 25μg/mL | 12.5μg/mL | CNCA |
| --- | --- | --- | --- | --- |
| 0 | 0±0 | 0±0 | 0±0 | 0±0 |
| 5 | 0.0026±0.0006 | 0.0029±0.0005 | 0.0032±0.0003 | 0.0036±0.0004 |
| 10 | 0.0052±0.0004 | 0.0051±0 | 0.006±0.0004 | 0.0066±0.0004 |
| 15 | 0.0072±0.0002 | 0.0066±0.0006 | 0.0084±0.0001 | 0.0096±0.0006 |
| 20 | 0.0091±0.0002 | 0.0082±0.0006 | 0.0112±0.0001 | 0.0143±0.0002 |

**Haval Unit**

| Days | 50μg/mL | 25μg/mL | 12.5μg/mL | CNCA |
| --- | --- | --- | --- | --- |
| 0 | 89.65±0.1838 | 88.57±0.6505 | 89.10±0.4455 | 89.65±0.2121 |
| 5 | 86.08±0.2404 | 82.94±1.0889 | 80.94±0.0778 | 77.60±0.8485 |
| 10 | 79.59±0.3677 | 76.13±1.4001 | 70.07±1.4779 | 68.00±0.7071 |
| 15 | 72.14±0.3677 | 70.37±0.0778 | 60.02±0.1768 | 57.50±0.7071 |
| 20 | 64.79±0.4455 | 60.73±0.8627 | 53.60±2.5597 | 47.15±0.9192 |

**Egg yolk index**

| Days | 50μg/mL | 25μg/mL | 12.5μg/mL | CNCA |
| --- | --- | --- | --- | --- |
| 0 | 0.45±0.0005 | 0.45±0.0017 | 0.46±0.0087 | 0.45±0.0012 |
| 5 | 0.42±0.0021 | 0.43±0.0081 | 0.41±0.0013 | 0.38±0.0088 |
| 10 | 0.40±0.0002 | 0.40±0.0042 | 0.36±0.0079 | 0.34±0.0118 |
| 15 | 0.38±0.0037 | 0.38±0.004 | 0.33±0.0042 | 0.27±0.0122 |
| 20 | 0.35±0.0048 | 0.34±0.0077 | 0.31±0.0092 | 0.26±0.0003 |

**Protein index**

| Days | 50μg/mL | 25μg/mL | 12.5μg/mL | CNCA |
| --- | --- | --- | --- | --- |
| 0 | 0.59±0.0038 | 0.57±0.0029 | 0.58±0.0007 | 0.57±0.0058 |
| 5 | 0.53±0.0036 | 0.53±0.0019 | 0.51±0.0006 | 0.47±0.0092 |
| 10 | 0.49±0.0037 | 0.48±0.0068 | 0.46±0.0049 | 0.44±0.0012 |
| 15 | 0.47±0.0118 | 0.45±0.0017 | 0.40±0.0034 | 0.41±0.0096 |
| 20 | 0.42±0.0003 | 0.40±0.0012 | 0.38±0.0038 | 0.35±0.0045 |

**Table S5. Data of the Evaluation of Different CCJY Concentrations on the Preservation Effect of Chilled Meat.**

**Drip Loss**

| Days | 50μg/mL | 25μg/mL | 12.5μg/mL | CNCA |
| --- | --- | --- | --- | --- |
| 0 | 0±0 | 0±0 | 0±0 | 0±0 |
| 1 | 0.30±0.1414 | 0.35±0.2121 | 1.15±0.2121 | 1.10±0.1414 |
| 2 | 1.65±0.495 | 1.95±0.0707 | 2.35±0.7778 | 1.90±0.1414 |
| 3 | 3.65±0.3536 | 2.75±0.7778 | 4.25±0.3536 | 4.45±0.0707 |
| 4 | 5.45±0.7778 | 4.15±0.3536 | 5.75±0.2121 | 6.25±0.0707 |
| 5 | 7.20±0.7071 | 6.60±0.4243 | 7.60±0.5657 | 8.50±0.7071 |
| 6 | 10.10±0.1414 | 8.90±0.1414 | 10.85±0.2121 | 11.70±0.4243 |
| 7 | 12.20±0.4243 | 11.00±0.1414 | 12.55±0.6364 | 13.10±0.1414 |
| 8 | 13.65±0.495 | 12.50±0.5657 | 14.00±0.1414 | 14.90±0.1414 |

**pH**

| Days | 50μg/mL | 25μg/mL | 12.5μg/mL | CNCA |
| --- | --- | --- | --- | --- |
| 0 | 5.20±0.1414 | 5.40±0.1414 | 5.50±0 | 5.40±0.1414 |
| 1 | 5.40±0.1414 | 5.50±0 | 5.60±0 | 5.60±0 |
| 2 | 5.45±0.0707 | 5.60±0 | 5.60±0 | 5.55±0.0707 |
| 3 | 5.65±0.0707 | 5.80±0 | 5.90±0 | 5.80±0 |
| 4 | 6.00±0 | 6.05±0.0707 | 6.00±0 | 6.65±0.0707 |
| 5 | 6.30±0 | 6.35±0.0707 | 6.50±0 | 6.95±0.0707 |
| 6 | 6.40±0.1414 | 6.50±0 | 6.75±0.0707 | 7.15±0.0707 |
| 7 | 6.70±0 | 7.05±0.0707 | 7.35±0.0707 | 7.50±0 |
| 8 | 7.05±0.0707 | 7.35±0.2121 | 7.75±0.0707 | 7.85±0.0707 |

**TBARS**

| Days | 50μg/mL | 25μg/mL | 12.5μg/mL | CNCA |
| --- | --- | --- | --- | --- |
| 0 | 0.18±0.0014 | 0.18±0.0049 | 0.19±0.0042 | 0.18±0.0049 |
| 1 | 0.19±0.0078 | 0.19±0.0021 | 0.20±0.0014 | 0.19±0.0021 |
| 2 | 0.24±0.0007 | 0.23±0.0035 | 0.25±0.0035 | 0.27±0.0049 |
| 3 | 0.25±0.0007 | 0.26±0.0035 | 0.29±0.0064 | 0.32±0.0212 |
| 4 | 0.27±0.0021 | 0.31±0.0085 | 0.36±0.0042 | 0.38±0.0057 |
| 5 | 0.32±0.0071 | 0.36±0.0064 | 0.42±0.0014 | 0.45±0.0035 |
| 6 | 0.37±0.0103 | 0.42±0.0035 | 0.48±0.0057 | 0.58±0.0057 |
| 7 | 0.43±0.0078 | 0.45±0.0028 | 0.55±0.0035 | 0.63±0.0021 |
| 8 | 0.48±0.0021 | 0.54±0.017 | 0.58±0.0057 | 0.68±0.0071 |

**TVB-N**

| Days | 50μg/mL | 25μg/mL | 12.5μg/mL | CNCA |
| --- | --- | --- | --- | --- |
| 0 | 7.6±0.5657 | 7.15±0.0707 | 7.75±0.495 | 7.55±0.495 |
| 1 | 8.65±0.2121 | 8.50±0.2828 | 9.15±0.0707 | 9.45±0.0707 |
| 2 | 9.35±0.2121 | 9.30±0.4243 | 10.60±0.1414 | 11.30±0.2828 |
| 3 | 10.85±0.0707 | 11.15±0.2121 | 12.80±0.1414 | 15.20±0.1414 |
| 4 | 12.90±0.2828 | 13.60±0.2828 | 15.25±0.0707 | 20.35±0.0707 |
| 5 | 15.50±0.2828 | 16.40±0.7071 | 19.30±0.2828 | 24.40±0.4243 |
| 6 | 18.65±0.495 | 22.15±3.3234 | 26.45±3.7477 | 30.45±0.3536 |
| 7 | 26.35±1.0607 | 25.90±3.1113 | 38.95±1.0607 | 42.35±3.182 |
| 8 | 39.30±0.7071 | 41.25±0.0707 | 48.30±3.9598 | 54.95±1.6263 |

**Reference**

1. Chen, G.-Z., Chumngoen, W., Kaewkot, C., Sun, Y.-M. and Tan, F.-J., Combination of sensory evaluation with conventional physiochemical analyses to evaluate quality changes during long-term storage and estimate the shelf life of chicken eggs. *Br. Poult. Sci.,* 2023, **64**, 594-604.

2. Katiyo, W., de Kock, H. L., Coorey, R. and Buys, E. M., Sensory implications of chicken meat spoilage in relation to microbial and physicochemical characteristics during refrigerated storage. *Lwt,* 2020, **128**, 109468.
